# Epidemiology, Clinical Profile, and Outcome of Acute Domestic Poisoning in Children in Four Health Facilities in the North West Region of Cameroon

**DOI:** 10.5152/eurasianjmed.2026.25885

**Published:** 2026-04-14

**Authors:** Andreas Chiabi, Kelly Reine Djoudja, Kate Kan, Joel Ngum Mbigha, Felicitas Sevidzem Lukong, Cecilia Fomenky, Loveline Lum Niba

**Affiliations:** 1 University of Bamenda Faculty of Health Sciences, Cameroun; 2 Regional Hospital, Bamenda, Cameroun; 3 Nkwen District Hospital, Bamenda, Cameroun; 4 Saint Blaise Catholic Hospital, Bamenda, Cameroun

**Keywords:** Acute domestic poisoning, children, clinical presentation, epidemiology

## Abstract

**Background:**

Acute intoxication is a series of pathophysiological changes or clinical manifestations over a short period (<24 hours) after exposure to toxic substances or toxic doses of drugs. Acute domestic poisoning is a preventable cause of morbidity and mortality among children, with a higher burden in low- and middle-income countries. This study aims to enhance the understanding of this public health problem and its complications, thereby promoting timely interventions, preventive strategies, and ultimately reducing mortality.

**Methods:**

A 5-year retrospective cohort study from 2019 to 2023 including 242 children aged 0-15 years hospitalized for acute domestic intoxication at Bamenda Regional Hospital, Nkwen District Hospital,Saint Mary Catholic Hospital and Saint Blaise Catholic Hospital.

**Results:**

Two hundred fifty-four cases of acute intoxication out of a total of 16 456 admissions were noted, giving a prevalence of 1.5% of admissions. Children were predominantly male (56.6%), with the 3-7 age group the most represented (33.1%). Toxic substances were mainly pesticides (19.0%), hydrocarbons (14.9%), and drugs (13.2%). Clinical manifestations were dominated by digestive (57.0%), neurological (34.3%), and respiratory (13.2%) symptoms. At home, the parents had given the child palm oil (46.3%) or charcoal (25.9%). A total of 9 deaths were recorded, representing a case-fatality rate of 4.2%, with a favorable clinical outcome in 95.8% of cases.

**Conclusion:**

Acute domestic poisoning remains frequent and a major pediatric emergency for children in the context. Parents are urged to keep toxic products out of their children’s reach and to seek appropriate medical attention in the event of intoxication.

**Main Points**

- Acute domestic poisoning is frequent among children in Bamenda, especially among boys under 7 years. Pesticides and hydrocarbons are the predominant toxic agents, and mortality remains high with pesticide and rodenticide exposure.
- Strengthening preventive education for caregivers, safe storage legislation, and early health-seeking behavior are critical to reducing related morbidity and mortality.

## Introduction

Acute poisoning refers to pathophysiological or clinical changes occurring within 24 hours following exposure to toxic substances or toxic doses of drugs.^1^ It is a major global public health issue, accounting for more than 10% of accidental injuries in children, with an incidence exceeding 280 cases per 100 000 inhabitants.^1^^,^^2^ According to the World Health Organization, approximately 350 000 people died from unintentional poisoning in 2020, with a mortality rate ranging from 3% to 6%, most in low- and middle-income countries.^3^ Nearly 50% of these cases involve children under the age of 6 years, who are particularly vulnerable.^4^

Children are especially at risk due to their natural curiosity and sensory exploration, including touching and tasting. Accidental poisoning predominantly affects children aged 12 years and under, whereas non-accidental poisoning becomes more frequent after this age.^5^ Acute domestic poisoning can lead to severe consequences, such as respiratory distress, heart failure, seizures, acute kidney injury, and even death. Prevention is crucial and can save lives if appropriate measures are taken to protect children.^6^^,^^7^

A study conducted in India reported that 56.8% of cases involved children under the age of 2 years, with a male predominance (66.7%). The main substances involved were petroleum products (45%), detergents (9%), and medications (8.1%), with intensive care unit hospitalization stays lasting between 1 and 3 days.^6^ In Egypt, the most commonly implicated substances were medications (analgesics, antipyretics), corrosive substances, and pesticides. While the majority of cases (75.8%) resulted in minor symptomatic effects, 3% of children died, primarily due to pesticide exposure.^4^ In Cameroon, data remain limited and geographically sparse. Most studies originate from Yaoundé or Limbe and reveal variations linked to local socioeconomic and agricultural practices.

The objective of this study was to analyze the epidemiology, clinical profile, and hospital outcomes of acute domestic poisoning among children in Bamenda. This study aimed to provide an understanding of this public health problem and its complications, thereby promoting timely interventions, preventive strategies, and ultimately reducing mortality. The hypothesis states that the prevalence of acute domestic poisoning in children in Bamenda is high, the most common substances involved in acute domestic poisoning will be kerosene, the clinical manifestation of acute domestic poisoning will be dominated by digestive symptoms, and the mortality rate of children with acute domestic poisoning will be high due to delayed or inappropriate management.

## Material and Methods

### Study Type and Period

This was a retrospective cohort study. It utilized the admission records of children hospitalized for acute domestic poisoning over a 5-year period (from January 1, 2019, to December 31, 2023). The study was conducted in 4 healthcare facilities within the Bamenda municipality which are the Regional Hospital Bamenda, Nkwen District Hospital, Saint Mary Catholic Hospital, and Saint Blaise Catholic Hospital that admit and hospitalize sick children. The current population of the Bamenda metropolitan area is approximately 615 000 inhabitants.

Acute domestic poisoning was defined as the occurrence of toxic exposure within the home environment—by ingestion, inhalation, or dermal absorption—leading to clinical manifestations within 24 hours. The study included all records of children aged 0 months to 15 years diagnosed with acute domestic poisoning. Excluded were cases of poisoning due to insect stings, animal bites, or records with incomplete essential data (e.g., child’s age, sex, or outcome). A total of 12 cases were excluded due to incomplete information in the file.

After obtaining ethical and administrative approvals, eligible cases were identified using a consecutive sampling technique from the medical records of children admitted to the selected hospitals for acute domestic poisoning. Informed consent from the participants was waived by the ethics committee. Records meeting the selection criteria were used to complete a data extraction form including socio-demographic characteristics, details of the poisoning event, clinical manifestation, severity assessed using the poisoning severity score (PSS),^8^ management received, and outcome.

Acute domestic poisoning is defined as a series of pathophysiological changes and relevant clinical manifestations in a short period (less than 24 hours) after ingestion, inhalation, or absorption of toxic substances within the home environment. The severity of poisoning was assessed using the PSS. Statistical analyses were performed using IBM SPSS Statistics for Windows, Version 26.0 (IBM SPSS Corp.; Armonk, NY, USA). Continuous variables are represented in means and standard deviation while categorical variables are expressed in frequencies and proportions. The chi-square test was used to assess the factors associated with acute domestic poisoning while multivariate logistic regression was used to adjust for confounders when assessing factors associated with acute domestic poisoning.

The study was approved by the Ethical Review Committee of the University of Bamenda Number 2024/0044H/UBa/IRB of April 5, 2024. Due to the retrospective nature of the study, informed consent requirement was waived by the ethics committee. Administrative approvals were granted by the North West Regional Delegation of Public Health and the directors of the participating hospitals.

## Results

A total of 16 456 children were admitted during the study period, of which 254 cases of acute domestic poisoning were identified. Twelve cases were excluded, resulting in a prevalence of 1.5%.

The age of the children ranged from 4 months to 15 years, with a mean age of 5.7 ± 4.3 years. The majority of cases occurred in children aged 3-7 years (33.1%). There were more boys (56.6%) than girls, yielding a sex ratio of 1.3. Additionally, most children were in primary school (37.6%) (Table 1).

**Figure 1.**
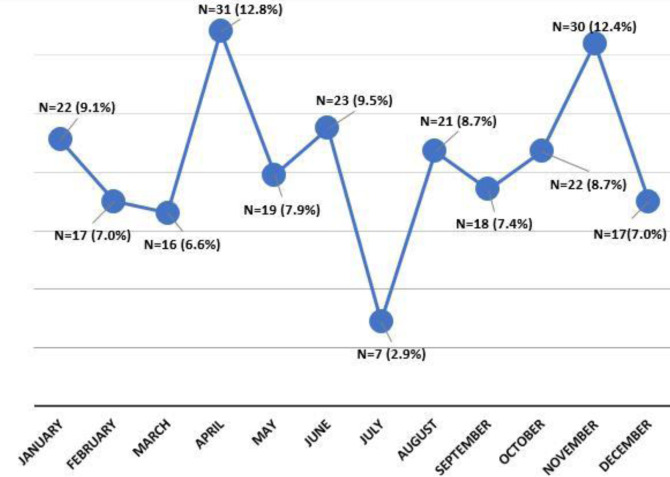
Monthly distribution of acute domestic poisoning cases.

The highest number of cases was recorded in April (12.8%) and November (12.4%), while the lowest rate was observed in July (2.9%) (Figure 1).

Pesticides were the most commonly ingested poisons (19.0%), followed by hydrocarbons (14.9%), medications (13.2%), and household cleaning products (11.6%) (Table 2).

In 47.1% of cases, the location of the incident was unknown. Among the known locations, most incidents occurred in the bedroom (31.4%), followed by the kitchen (14.0%) and the school (4.5%). The majority of incidents were accidental (86.8%) and occurred in the afternoon (64.1%) while the child was alone (81.0%).

Among the 242 participants, 190 (78.5%) presented with symptoms, 48 (19.8%) were asymptomatic, while 4 (1.7%) died before consultation. Regarding clinical presentation, 78.5% presented symptomatically. Digestive manifestations (57.0%), mainly vomiting, were most frequent, followed by neurological (34.3%), respiratory (13.2%), and cardiovascular signs (11.6%) (Table 3). Based on the PSS, most cases were minor or moderate; 4 cases (1.7%) were severe.

Before arriving at the hospital, caregivers commonly administered palm oil (46.3%) or charcoal (25.9%). In-hospital management included activated charcoal (58.8%) and gastric lavage (23.1%). Most children recovered favorably, with 95.8% discharged home. Nine deaths occurred, giving a case-fatality rate of 4.2% (Table 4).

On multivariate analysis, individuals who ingested more than 1 toxic substance (OR = 11.06; 95% CI (2.37-51.49); *P* = .002), ingested pesticides (OR = 11.88; 95% CI (2.82-50.03); *P* = .001), or ingested rat poison (OR = 14.36; 95% CI (2.97-69.53); *P* = .001) were more likely to die compared to those who ingested only 1 substance (Table 5).

After adjusting for confounding factors, the ingestion of pesticides (aOR = 14.99; 95% CI (2.59-86.86); *P* = .003) and rat poison (aOR = 19.49; 95% CI (1.87-203.19); *P* = .013) was found to significantly increase the risk of death.

## Discussion

Acute domestic poisonings primarily affected children aged 3 to 7 years, with a mean age of 5.7 ± 4.3 years. The predominance among children below 7 years aligns with findings from Ethiopia and China, supporting the theory of high exploration behavior, inadequate supervision in this age group, and limited understanding of dangers.^9^^,^^10^ The male predominance is similarly supported by numerous multi-regional studies attributing this to gender-based risk-taking tendencies and curious behavior, which increases the risk of accidental poisoning.^9^^,^^11^

The prevalence of acute poisoning was 1.5%, similar to the 1.5% reported in Ethiopia and 1.6% in Yaoundé.^9^^,^^12^ However, it was higher than the 0.28% reported in Nigeria and 1.14% in Nepal,^13^^,^^14^ a discrepancy that may be related to differences in substance storage practices, education, or regulatory measures. The monthly distribution of cases showed peaks in April and November, with the lowest incidence in July. These variations may be explained by seasonal activities, such as increased pesticide use during agricultural periods common in Sub-Sahara Africa.^9^^,^^15^

Pesticides were the leading toxic agents (19.0%), in line with findings from other studies, particularly in agricultural regions like the north west Cameroon.^9^^,^^16^^-^^18^ However, in Yaounde, hydrocarbons were the most common toxic substances,^10^^,^^19^ a difference likely attributable to the prominence of agriculture in the North West region of Cameroon, facilitating pesticide accessibility. The most common time of ingestion was in the afternoon, possibly because children are more active and less supervised at that time, increasing their risk of exposure.^19^^,^^20^

In the study, gastrointestinal symptoms, particularly vomiting, were the most frequent clinical manifestations upon admission. This is likely because most of the toxic substances were ingested through the oral route or because of the side effects of certain chemicals.^10^ Most cases of domestic poisoning were classified as asymptomatic (19.9%), mild (35.1%), or moderate (29.3%), indicating that the majority were not life-threatening and could be managed medically with a good recovery without sequelae. However, the presence of a small percentage (15.7%) of severe or fatal cases was life-threatening, highlighting the importance of prompt intervention.

Regarding treatments before consultation, palm oil and charcoal were the most commonly administered home remedies, whereas other studies have reported milk as the primary home remedy. These substances are often used based on traditional beliefs or misinformation.^19^

The majority of children were discharged from the hospital after complete recovery, confirming the effectiveness of prompt medical care. However, 4.2% of cases were fatal; this is higher than the 0.3% reported in Egypt^4^ but close to the 3.2% observed in Yaoundé^10^. This difference may be attributed to the type of toxic substances ingested, the availability and access to healthcare, environmental factors, and cultural practices influencing responses to poisoning incidents.

Ingestion of more than 2 substances increased the risk of death by 11-fold, suggesting a probable additive or synergistic interaction between toxins. Pesticides and rat poisons increased the risk of mortality by 11 and 14 times respectively, due to their high toxicity (e.g., brodifacoum, organophosphates), which can cause fatal hemorrhages or respiratory failure even at low doses. Multivariate logistic regression was done after controlling for the variables rat poison, pesticides, and number of toxins ingested, confirming that exposure to these substances (rat poison and pesticides) was an independent predictor of mortality, as corroborated by other authors.^9^ These findings highlight the critical role of toxin type in determining outcomes of domestic poisonings in children.

The main limitation of the study was the retrospective design, which relied on retrospective data and might have introduced reporting bias or incomplete data, also limiting the verification of the accuracy of the recorded details. The inability to capture long-term complications or community-level unreported cases, as well as the exclusion of incomplete records for missing essential data, might have led to underestimation of the prevalence and outcomes.

Acute domestic poisonings remain a significant concern in the setting, predominantly affecting young children. The most frequently implicated substances were pesticides, hydrocarbons, and medications. Clinical manifestations were primarily gastrointestinal, neurological, and respiratory symptoms. The mortality rate remains high, underscoring the need for increased parental vigilance to prevent accidental poisonings at home.

## Figures and Tables

**Table 1.** Sociodemographic Characteristics of Children (n = 242)

**Variables**	**Number (n = 242)**	**Percentage (%)**
Age (years)		
<1	9	3.7
(1-3)	66	27.3
(3-7)	80	33.1
(7-11)	49	20.2
(11-15)	38	15.7
Sex		
Male	137	56.6
Female	105	43.4
Level of education		
Pre school	80	33.1
Nursery	35	14.5
Primary	91	37.6
Secondary	36	14.9
Place of residence		
Urban	185	76.4
Rural	57	23.6

**Table 2.** Types of Ingested Poisons (n = 242)

**Type of Poison**	**Number (N = 242)**	**Percentage (%)**
Pesticides	46	19.0
Hydrocarbons 36 (14.9%)		
Petroleum	28	77.8
Essence	7	19.4
Diesel	1	2.8
Medicines 32 (13.2%)		
Analgesics	8	25.0
Antihypertensives	8	25.0
Antidiabetics	4	12.5
Antiepileptics	4	12.5
Digitalis	1	3.1
Others*	7	21.9
Household products 28 (11.6%)		
Caustic soda	12	42.9
Bleach	9	32.1
Detergents	6	21.4
Polish	1	3.6
Cosmetic products	19	7.9
Plants	18	7.4
Contaminated food	12	4.9
Alcohol	11	4.5
Rat poison	11	4.5
Illicit drugs	10	4.1
Unknown	6	2.5
Others**	13	5.3

*Vitamins, calcium, antidepressants, antiemetics and **Coins, batteries, mushrooms, pen ink.

**Table 3.** Clinical Manifestations by Affected System

**System**	**Numbers (n)**	**Percentage (%)**
Digestive system: 138 (57%)		
Vomiting	113	81.9
Abdominal pain	67	48.5
Sialorrhea/excessive salivation	53	38.4
Diarrhoea	26	18.8
Others	10	7.2
Nervous system: 83 (34.3%)		
Agitation	59	71.0
Seizure	30	36.1
Loss of consciousness	15	18.1
Myosis	2	2.4
Others	22	26.5
Respiratory system: 32 (13.2%)		
Dyspnea	20	62.5
Cough	17	53.1
Chest pain	4	12.5
Others	4	12.5
Cardiovascular system	28	11.6
Integumentary system	17	7.0
Urogenital system	4	1.6

**Table 4.** Extra- and Intra-Hospital Management

**Treatment **	**Number (n)**	**Percentage (%)**
Treatment at home before consultation		
Palm oil	50	46.3
Charcoal	28	25.9
Milk	13	12.0
Honey	13	12.0
Africa panacea	2	1.9
Bitter cola	1	0.9
Olive oil	1	0.9
None	134	55.4
Hospital treatment		
Activated charcoal	107	58.8
Gastric lavage	42	23.1
Oxygen therapy	18	9.9
Antidotes	8	4.4
Surgery*	1	0.5

*Laparotomy indicated for battery extraction.

**Table 5.** Predictive Factors of Mortality in Acute Household Poisoning (n = 217)*

**Variables**	**Death**		**Survived**		**RC (95% CI)**	*P*
	**(n = 9)**	**(%)**	**(n = 208)**	**(%)**		
Length of hospital stay						
1	1	11.1	38	18.3	0.13 (0.01-1.60)	.112
4-6	6	66.7	46	22.1	0.65 (0.11-3.72)	.630
>6	2	22.2	10	4.8	Reference	
Poisoning severity score						
Stage 3	2	22.2	61	29.3	Reference	
Stage 4	3	33.3	22	10.6	4.16 (0.65-26.57)	.132
Age at the time of diagnosis						
(1-4)	2	22.2	84	39.3	0.38 (0.05-2.82)	.345
(4-7)	3	33.3	43	21.1	1.12 (0.18-7.01)	.907
(7-10)	2	22.2	41	19.0	0.78 (0.10-5.85)	.809
(10)	2	22.2	32	16.9	Reference	
Pesticides	6	66.7	30	14.4	**11.88 (2.82-50.03)**	**.001**
Hydrocarbons	1	11.1	31	14.9	0.714 (0.09-5.91)	.754
Rat poison	3	33.3	7	3.4	**14.36 (2.97-69.53)**	**.001**
Number of toxins ingested						
1	6	66.7	199	95.7	Reference	
≥2	3	33.3	9	4.3	**11.06 (2.37-51.49)**	**.002**

The bold was for significant *P* Value that is a *P* value < 0.05. CI; confidence interval.

*13 were discharged against medical advice, and 12 were referred to other health facilities.

## Data Availability

The data that support the findings of this study are available on request from the corresponding author.

## References

[1] Li Z Xiao L Yang L Characterization of acute poisoning in hospitalized children in Southwest China. Front Pediatr. 2021;9:727900. (doi: 10.3389/fped.2021.727900) PMC8705540 34956970

[2] Peden M Oyegbite K Ozanne-Smith J World rReport on cChild iInjury pPrevention. New York: UNICEF; 2008.

[3] World Health Organization. World Report on Child Injury Prevention. Geneva: WHO; 2008. Available from: https://iris.who.int/handle/10665/43851. 26269872

[4] Azab SMS Hirshon JM Hayes BD Epidemiology of acute poisoning in children presenting to the poisoning treatment center at Ain Shams University in Cairo, Egypt, 2009-2013. Clin Toxicol (Phila). 2016;54(1):20 26. (doi: 10.3109/15563650.2015.1112014) 26653953 PMC4933840

[5] Alwan IA Brhaish AS Awadh AI Poisoning among children in Malaysia: a 10-year retrospective studyPoisoning among children in Malaysia: a 10-years retrospective study. PLoOS One. 2022;17(54):e0266767. (doi: 10.1371/journal.pone.0266767) PMC9049302 35482773

[6] Rameshkumar NS Tamilarasan P Arunagirinathan A. Accidental household poisoning in children: shedding light on the common agents and risk factors. Int J Contemp Pediatr. 2021;8(79):1522 1527. (doi: 10.18203/2349-3291.ijcp20213313)

[7] Zemedie B Sultan M Zewdie A. Acute poisoning cases presented to the Addis Ababa burn, emergency, and trauma hospital emergency department, Addis Ababa, Ethiopia: a cross-sectional study. Emerg Med Int. 2021;2021:6028123. (doi: 10.1155/2021/6028123) PMC8674071 34925919

[8] Heath A.. Laboratory diagnosis of acute poisoning: Cconsequences for treatment. In: den Boer NC , van der Heiden C , Leijnse B , Souverijn JHM . Clinical cChemistry overview. US. Boston (MA): Springer US; 1989:p. 103 109. (doi: 10.1007/978-1-4613-0753-2_11)

[9] Dai Q Wang L Gao X Clinical and epidemiological characteristics of acute poisoning in children in South western China: a review of 1755 cases from 2014 to 2020. Int J Gen Med. 2022;15:133 142. (doi: 10.2147/IJGM.S342253) 35027838 PMC8749043

[10] Bengono Bengono RS Amengle AL Mbengono Metogo JA Intoxications aiguës aux urgences pédiatriques de l’hôpital gynéco-obstétrique et pédiatrique de Yaoundé (Cameroun). Anesth Reanim. 2021;7(5):330 336. (doi: 10.1016/j.anrea.2021.06.009)

[11] Kenko DBN Mekone AE Ekaineck JM. Trends in poisoning and bites among patients referred to the Limbe Regional Hospital, South-West Cameroon. Eur Sci J. 2021;17(3):104 120.

[12] Molla YM Belachew KD Ayehu GW Acute poisoning in children in Ethiopia: a cross-sectional study. Sci Rep. 2022;12(1):18750. (doi: 10.1038/s41598-022-23193-x) PMC9637174 36335242

[13] Isaac E Jalo I Adamu S Spectrum of poisoning and outcome among children in a tertiary hospital, North-East Nigeria: a 20-year retrospective review, 2000-2019. Open J Pediatr. 2022;12(3):100 124.

[14] Aryal S Karki S Lamichhane M. Acute poisoning among children admitted in a tertiary care hospital: a descriptive cross-sectional study. JNMA J Nepal Med Assoc.. 2024;62(25271):160 164. (doi: 10.31729/jnma.8482) 39356792 PMC10924476

[15] Ansong D Nkyi CA Appiah C Epidemiology of pediatric poisoning reporting to a tertiary hospital in Ghana. Sci J Clin Med.. 2016;10(2):68 74.

[16] Malangu N.. Acute poisoning at two hospitals in Kampala-Uganda. J Forensic Leg Med. 2008;15(8):489 492. (doi: 10.1016/j.jflm.2008.04.003) 18926499

[17] Zhang H Huo Q Jing R Clinical analysis of acute poisoning in children. BMC Pediatr. 2024;24(1):212. (doi: 10.1186/s12887-024-04697-z) PMC10962155 38528509

[18] Malangu N Ogunbanjo GA. A profile of acute poisoning at selected hospitals in South Africa. S Afr J Epidemiol Infect. 2009;24(2):14 16. (doi: 10.1080/10158782.2009.11441343)

[19] Nguefack F Chiabi A Ndounia SN Clinical and epidemiologic study on unintentional domestic poisoning at the paediatric service of the Yaounde Gynaeco-Obstetric and Pediatric Hospital. J Med Res. 2017;3(3):164 168. (doi: 10.31254/jmr.2017.3314)

[20] Nguyen SN Vu LT Nguyen HT Childhood acute poisoning at Haiphong Children’s Hospital: a 10-year retrospective study. Int J Pediatr. 2023;2023:2130755. (doi: 10.1155/2023/2130755) PMC10495236 37700774

